# A Vitronectin-Derived Bioactive Peptide Improves Bone Healing Capacity of SLA Titanium Surfaces

**DOI:** 10.3390/ma12203400

**Published:** 2019-10-17

**Authors:** Chang-Bin Cho, Sung Youn Jung, Cho Yeon Park, Hyun Ki Kang, In-Sung Luke Yeo, Byung-Moo Min

**Affiliations:** 1 Department of Prosthodontics, Seoul National University School of Dentistry, and Dental Research Institute, 101 Daehak-Ro, Jongno-Gu, Seoul 03080, Korea; istardental@naver.com; 2 Department of Oral Biochemistry and Program in Cancer and Developmental Biology, Seoul National University School of Dentistry, and Dental Research Institute, 101 Daehak-Ro, Jongno-Gu, Seoul 03080, Korea; jsy1618@snu.ac.kr (S.Y.J.); choyeon94@snu.ac.kr (C.Y.P.); kang1978@snu.ac.kr (H.K.K.)

**Keywords:** vitronectin, RVYFFKGKQYWE motif, cellular responses, dental implants, osseointegration

## Abstract

In this study, we evaluated early bone responses to a vitronectin-derived, minimal core bioactive peptide, RVYFFKGKQYWE motif (VnP-16), both in vitro and in vivo, when the peptide was treated on sandblasted, large-grit, acid-etched (SLA) titanium surfaces. Four surface types of titanium discs and of titanium screw-shaped implants were prepared: control, SLA, scrambled peptide-treated, and VnP-16-treated surfaces. Cellular responses, such as attachment, spreading, migration, and viability of human osteoblast-like HOS and MG63 cells were evaluated in vitro on the titanium discs. Using the rabbit tibia model with the split plot design, the implants were inserted into the tibiae of four New Zealand white rabbits. After two weeks of implant insertion, the rabbits were sacrificed, the undecalcified specimens were prepared for light microscopy, and the histomorphometric data were measured. Analysis of variance tests were used for the quantitative evaluations in this study. VnP-16 was non-cytotoxic and promoted attachment and spreading of the human osteoblast-like cells. The VnP-16-treated SLA implants showed no antigenic activities at the interfaces between the bones and the implants and indicated excellent bone-to-implant contact ratios, the means of which were significantly higher than those in the SP-treated implants. VnP-16 reinforces the osteogenic potential of the SLA titanium dental implant.

## 1. Introduction

Attachment of cells is the first step in cell–biomaterial interactions [1]. The transmembrane proteins on the cell membrane recognize and bind biomacromolecules adsorbed on the surface of the biomaterial [1]. These biomacromolecules are from the extracellular matrix (ECM), controlling cellular behaviors such as attachment, spreading, proliferation, and differentiation depending on the interacting transmembrane receptors [2]. Therefore, these ECM biomolecules, if applied to the titanium dental implant surface, are anticipated to have potential in reinforcing osseointegration [3,4,5]. One candidate molecule is vitronectin.

Vitronectin, one of the ECM proteins, is an abundant multifunctional glycoprotein found in serum, the extracellular matrix, and bone, and is involved in various physiological processes such as cell attachment, spreading, and migration [6,7,8]. Vitronectin contributes to healing of the bone surrounding a dental implant by promoting the attachment and spreading of the osteogenic cells [9,10]. This ECM protein appears to play a major role in initial bone healing by reorganizing the intracellular microfilaments and microtubules, which facilitates cell attachment and spreading [11,12,13]. However, the use of such an original protein has several critical limitations: a high cost for synthesis, antigenicity and instability of the molecule, and steric hindrance of this macromolecule in focal adhesion [5,14,15]. A functional peptide derived from the parent protein is a notable alternative, overcoming these limitations and maintaining the original biological activity [3,5,9,14,16,17]. In addition, bioactive peptides have advantages over larger protein molecules due to their robustness and sterilizability [18]. Recently, a vitronectin-derived functional peptide sequence, RVYFFKGKQYWE (VnP-16), has shown a Janus regulation for bone formation: promotion of osteoblast activity and inhibition of osteoclast activity, which is a desirable effect for osteogenesis that vitronectin does not have [9]. The VnP-16 peptide promoted bone formation by accelerating osteoblast differentiation and activity through direct interaction with β1 integrin followed by focal adhesion kinase (FAK) activation. Concomitantly, the peptide inhibited bone resorption by restraining Janus N-terminal Kinase (JNK)-c-Fos-nuclear factor of activated T cells, cytoplasmic 1 (NFATc1)-induced osteoclast differentiation and αvβ3 integrin-c-Src-proline-rich tyrosine kinase 2 (PYK2)-mediated resorptive function. Moreover, VnP-16 peptide decreased the bone resorbing activity of pre-existing mature osteoclasts without changing their survival rate [9].

The functionalization of a titanium implant via the immobilization of desirable proteins or their bioactive peptides in their native conformations is a promising approach to overcome the bioinertness of the surface, leading to improved osseointegration [18,19]. Several methodologies, including physical adsorption, covalent immobilization via chemical methods, and covalent immobilization via physical methods, have been investigated since the inception of protein functionalization on titanium substrates [19]. Physical adsorption is the simplest method to immobilize proteins on titanium substrates. Chemical immobilization, the most established of the protein functionalization approaches, proved that it was possible to covalently immobilize proteins to titanium substrates, overcoming the unintentional protein release observed in adsorption approaches. Physical covalent immobilization of biomolecules is the most recent approach to dental and orthopedic biomimetic functionalization, and possesses advantages over adsorption and chemical covalent immobilization [19]. Other methods on peptide-functionalization of titanium surfaces are also reported. Control of both peptide orientation and surface concentration is achieved by varying the solution pH or by applying an electric field [18]. In addition, multifunctional coating improves cell adhesion on titanium surfaces by using cooperatively acting peptides [20].

A micro-roughened surface of commercially pure titanium has been clinically used in the field of implant dentistry [10]. A sandblasted large-grit acid-etched (SLA) surface with approximately 1.5 μm of arithmetic mean deviation is known to accelerate osseointegration at the bone-implant interface, compared to the turned surface with no modification at the micro-level [10,21,22]. The application of VnP-16 to the SLA surface increases clinical relevance in the further enhancement of bone formation and makes use of dental implants extended to patients suffering from bone metabolic weaknesses, like osteoporosis. The VnP-16-treated SLA titanium surface has not been investigated yet.

This study aimed to evaluate the early bone response to the VnP-16-treated SLA titanium surface in vivo. In vitro tests were also performed using osteoblast-like cells. The hypothesis underlying this study was that the application of VnP-16 would further reinforce the osteogenic potential of the SLA surface.

## 2. Materials and Methods

### 2.1. Cells, Peptides, and Reagents

HOS and MG-63 cells, lines derived from human osteosarcomas, were purchased from the American Type Culture Collection (Rockville, MD, USA) and cultured in Dulbecco’s modified Eagle’s medium (Gibco BRL, Carlsbad, CA, USA) supplemented with 10% fetal bovine serum. Each peptide was synthesized using the 9-fluorenylmethoxycarbonyl-based solid-phase method with a C-terminal amide using a Pioneer Peptide Synthesizer (Applied Biosystems, Foster City, CA, USA) in the Peptron (Daejeon, Korea). The synthetic peptides used in the study had a purity greater than 95%, as determined using high-performance liquid chromatography. Human plasma vitronectin was obtained from Millipore (Bedford, MA, USA).

### 2.2. Disc Preparation and Surface Characterization

Titanium disc specimens, which were 0.5 mm thick and 10 mm in radius, were made of commercially pure grade 4 titanium. The discs, serving as control, were prepared by polishing with #600 and #1200 sandpaper. The other discs were subjected to sandblasting with large alumina particles and etched with a hydrochloric acid solution to generate the SLA surface (Deep Implant System, Seongnam, Korea). The SLA titanium discs were rinsed, ultrasonically washed, and dried. One group of the SLA discs was left untreated, another was treated with a scrambled peptide (SP; 10.5 μg/cm^2^), and the other was treated with VnP-16 (10.5 μg/cm^2^).

The surfaces of the four types of disc were imaged using field emission-scanning electron microscopy (FE-SEM; S-4700, Hitachi, Tokyo, Japan). The element composition of each group was analyzed by electron spectroscopy for the chemical analysis (ESCA; Sigma Probe, Thermo Scientific, Waltham, MA, USA). Confocal laser scanning microscopy (CLSM; LSM 800, Carl Zeiss AG, Oberkochen, Germany) calculated two surface parameters for surface topography of the investigated discs; arithmetic mean deviation (Ra) and the developed surface area ratio (Sdr) [23].

### 2.3. Cell Attachment and Spreading Assays

The cell attachment assay was performed as described previously [9]. The physical adsorption method was used for the application of peptides. Twenty-four-well culture plates were coated with 0.26 μg/cm^2^ human plasma vitronectin for 24 h at 4 °C or 10.5 μg/cm^2^ synthetic peptides for 24 h at room temperature, blocked with 1% heat-inactivated bovine serum albumin (BSA) in phosphate-buffered saline (PBS) for 1 h at 37 °C, and then washed with PBS. Cells (1 × 10^5^ cells/500 μL) were added to each plate and incubated in serum-free culture medium for 1 h at 37 °C. After incubation period, unattached cells were removed by rinsing twice with PBS. Attached cells were fixed with 10% formalin for 15 min, stained with 0.5% crystal violet for 1 h, gently washed with distilled water three times, and dissolved with 2% sodium dodecyl sulfate for 5 min. Absorbance was measured at 570 nm using a microplate reader. For cell-spreading assay, cells (7 × 10^4^ cells/500 μL) were added to each substrate-coated plate and incubated for 3 h at 37 °C. To determine cell spreading, formalin-fixed and crystal violet-stained cell surface area was measured with Image-Pro plus software (Version 4.5; Media Cybernetics, Silver Spring, MD, USA).

### 2.4. Migration Assay

Migration assays were performed using a transwell migration chamber (Corning, Pittston, PA, USA) possessing 8 μm pores as described previously [24]. The lower side of each transwell filter was coated with vitronectin (0.26 μg/cm^2^), or synthetic peptides (10.5 μg/cm^2^) by drying for 24 h at 4 °C (vitronectin) or for 24 h at room temperature (peptides). Cells (2 × 10^4^ cells/24-well) were seeded in the upper chamber of a transwell filter and allowed to migrate for 24 h at 37 °C. Cells were then fixed with 10% formalin for 15 min and stained with 0.5% crystal violet. Unmigrated cells in the upper side of the transwell filter were removed with a cotton swab, and cell migration was quantified by counting the number of cells that had migrated through the filter. Human placental laminin was used as the positive controls and SP was used as the negative control.

### 2.5. Cell Viability Assay

The viabilities of cells were investigated using the EZ-Cytox Cell Viability Assay kit (water-soluble tetrazolium salt method; Daeil Lab Service, Seoul, Korea). A 96-well microplate was coated with VnP-16 peptide (0, 10.6, 21.2, or 42.4 μg/cm^2^) by drying for 24 h at room temperature. Cells (1.5 × 10^4^ cells/100 μL) were seeded onto a 96-well microplate and then cultured for 24 h or 48 h at 37 °C. The water-soluble tetrazolium salt reagent solution (10 μL) was added to each well, and the plate was incubated for 2 h at 37 °C. The absorbance at 450 nm was then measured using a microplate reader.

### 2.6. In Vivo Experiment

Sixteen screw-shaped grade 4 titanium implants were made, which were 3.5 mm in major diameter and 11 mm in length (Warantec, Seongnam, Korea). Four implants were used as they were without any surface modification and designated as turned surface (control). The surface of another four implants was SLA (Deep Implant System, Seongnam, Korea). Half of the rest of the eight implants were treated with SP while the other half were treated with VnP-16 (1.0 mg/cm^2^). Using the physical adsorption method, the Ti implants were placed on 0.2 ml PCR tubes and coated with the synthetic peptides by drying for 7 d in a vacuum at room temperature.

All the animal experiments performed in this study were approved by the Ethics Committee of Animal Experimentation of the Institutional Animal Care and Use Committee (CRONEX-IACUC 201705001; Cronex, Hwasung, Korea). These experiments were conducted following the Animal Research: Reporting In Vivo Experiments (ARRIVE) guidelines for the care and use of laboratory animals [25]. Four New Zealand white rabbits were used in this study, which were male, approximately five to six months in age and 2.5 to 3.0 kg in weight. The experimental animals were intramuscularly anesthetized with a dose of 15 mg/kg tiletamine hydrochloride and zolazepam hydrochloride (Zoletil, Virbac, Carros, France) and 5 mg/kg xylazine (Rompun, Bayer AG, Leverkusen, Germany). The skin hair was shaved at the tibial area of the rabbits, which was disinfected with aqueous iodine. A full-thickness incision from the skin to the periosteum of the tibiae was made, and the flaps were elevated to expose the medial surfaces of the tibiae. Drilling was conducted for to make the holes on the medial surfaces for implant insertion. The final diameter of the holes was 3.2 mm. Two implants were inserted into each tibia and arranged according to the split plot design. The periosteum and fascia were sutured with 4-0 polyglactin 910 (Vicryl, Ethicon, Somerville, NJ, USA) while the skin was sutured with 4-0 Nylon (Ethilon, Ethicon, Somerville, NJ, USA). Each experimental animal was housed in a separate cage and an antibiotic, enrofloxacin, (Biotril, Komipharm International, Siheung, Korea) was administered to prevent infection.

### 2.7. Light Microscopic Evaluation

The rabbits were sacrificed under general anesthesia with the intravenous administration of potassium chloride at 14 days after implant insertion. The implants were removed en bloc with the surrounding bones and fixed in 10% neutral buffered formalin for 2 weeks. After formalin fixation, each implant-bone block was dehydrated with ethanol. Then, the blocks were resin-embedded (Technovit 7200, Heraeus Kulzer, Hanau, Germany) and ground for light microscopy using an EXAKT system (EXAKT Apparatebau, Norderstedt, Germany), according to the methods described in the previous studies [23,26]. Sections of the implant-bone blocks were prepared with a final thickness of approximately 50 μm and modified Goldner’s Masson trichrome staining [27]. The interfacial areas between the bones and implants were observed and evaluated from the bone crests to 2 mm in depth for histomorphometry, where bone-to-implant contact (BIC) and bone area (BA) ratios were calculated. The image analyses and the histomorphometric calculations were performed on ×100 magnified images using a light microscope (BX51, Olympus, Tokyo, Japan), SPOT version 4.0 software (Diagnostic Instruments, Sterling Heights, MI, USA) and Image-Pro Plus (Media Cybernetics, Rockville, MD, USA).

### 2.8. Statistics

Descriptive statistics for the data were presented as the mean ± standard deviation (SD). The statistical analyses were performed with R software (version 3.6.1, R Foundation for Statistical Computing, Vienna, Austria). All the data obtained in this study were confirmed to be normally distributed by the Shapiro–Wilk test. Analysis of variance tests were used for comparisons among the groups. When a significant difference was found, Tukey’s honestly significant difference test was further applied for pairwise comparison. The level of significance was 0.05 in this study.

## 3. Results

### 3.1. Surface Characteristics

**Figure 1:**
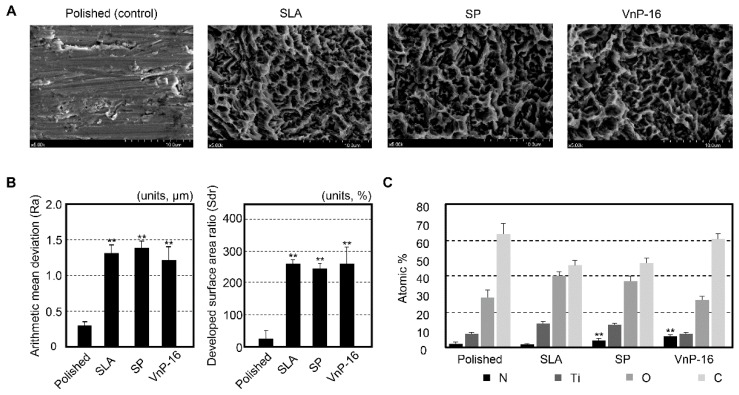
Surface characteristics of the titanium specimens investigated in this study. (**A**) Field emission scanning electron microscopy definitely shows different topographical features between the polished and sandblasted, large-grit, acid-etched (SLA) surfaces. (**B**) The mean values of the measured surface parameters indicated that the peptide treatment did not change the surfaces physically at the micro level. Note the significant differences in the surface parameters between the polished and the other SLA surfaces. ^**^ *p* < 0.01 vs. the polished surface. (**C**) Electron spectroscopy for chemical analysis detected high nitrogen content on the peptide-treated surfaces. Almost no nitrogen was found in the other groups. ^**^ *p* < 0.01 vs. the polished and SLA surfaces (significant differences are marked only for the nitrogen content).

The FE-SEM images of the specimens showed very different topographical features between the polished and SLA surfaces (Figure 1A). Some grooves on the overall flat surfaces were found for the polished specimens, while a honeycomb-like irregular topography was observed for the SLA specimens. The treatments of SP and VnP-16 had little effect on the surface physical features of the specimens (Figure 1A). There was no significant difference in either the Ra or Sdr among the SLA titanium discs, regardless of the peptide treatment (*p* > 0.05) (Figure 1B). However, in surface chemistry, the treatments of the functional peptides were confirmed from the results of higher nitrogen contents for the SP- and VnP-16-treated surfaces, compared to those for the other groups (polished and SLA titanium surfaces) (*p* < 0.05) (Figure 1C). The highest content element was carbon for every group.

### 3.2. Effects of VnP-16 Peptide on Cellular Responses of Human Osteoblast-Like Cells

**Figure 2:**
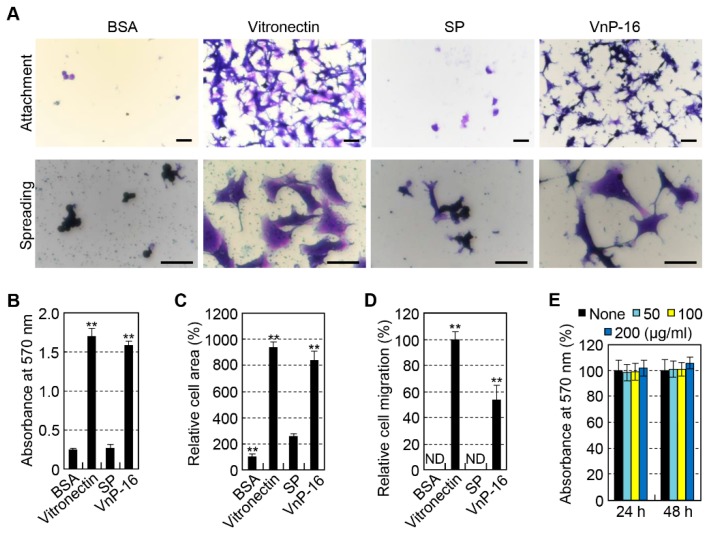
Cell attachment, spreading, and migration of osteoblast-like HOS cells seeded on culture plates treated with vitronectin and synthetic peptides. (**A**) Photographs of osteoblast-like HOS cells adhering (upper panel) and spreading (lower panel) to culture plates treated with 1% bovine serum albumin (BSA), vitronectin (0.26 μg/cm^2^), scrambled peptide (SP), and VnP-16 peptide (10.5 μg/cm^2^). Bar = 100 μm. (**B**,**C**) Cell attachment (**B**) and spreading (**C**) to immobilized synthetic peptides. HOS cells were allowed to adhere to peptide-treated plates for 1 h (**B**) or 3 h (**C**) in serum-free medium. (**D**) Migration of osteoblast-like HOS cells induced by vitronectin and synthetic peptides. HOS cells were seeded into the upper chambers of transwell filters coated with vitronectin (0.26 μg/cm^2^), SP, or VnP-16 (10.5 μg/cm^2^) and were incubated for 24 h. ND, not detected. (**E**) The viabilities of osteoblast-like HOS cells treated with VnP-16 for 24 or 48 h. ^**^ *p* < 0.01 vs. the SP-treated control group. Data in (**B**–**E**) (*n* = 4) represent the mean ± SD.

To investigate whether a human vitronectin-derived peptide, VnP-16, could mediate cell behavior of osteoblasts, cell attachment, spreading, and migration of human osteoblast-like cells, including HOS and MG-63, were assayed. The attachment of osteoblast-like cells was evaluated using a cell adhesion assay in a serum-free medium. Human plasma vitronectin strongly promoted cell attachment (Figure 2A upper, B) and spreading (Figure 2A lower, C) in osteoblast-like HOS cells. The VnP-16 peptide also promoted greater cell attachment (Figure 2A upper, B) and spreading (Figure 2A lower, C) than the BSA or SP control, and its attachment and spreading activities were comparable to those of vitronectin (Figure 2A–C). In addition, vehicle and SP did not participate in cell migration in HOS cells. On the other hand, vitronectin and the VnP-16 peptide promoted cell migration in HOS cells, while the VnP-16 peptide was significantly less effective than vitronectin (Figure 2D). The VnP-16 peptide did not affect the proliferation or viability of HOS cells (Figure 2E), indicating that its stimulatory effect on the cell behavior of HOS cells was not due to cytotoxicity or enhanced cell proliferation. These results support that VnP-16 is functionally active in promoting osteoblastic responses.

**Figure 3:**
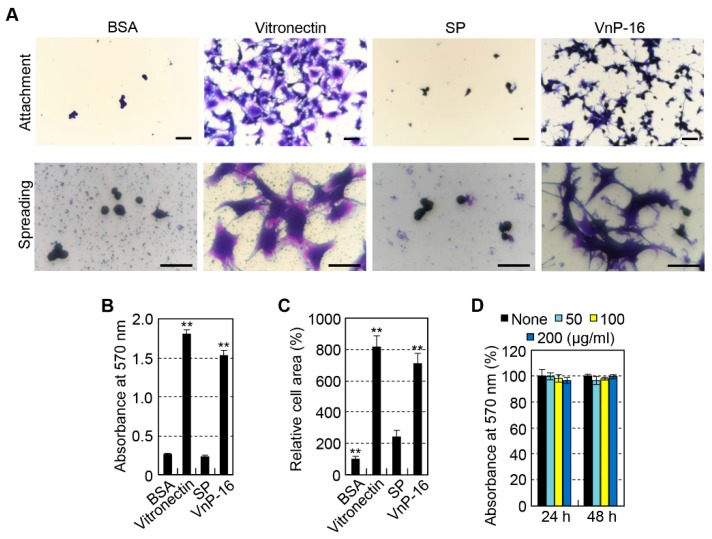
Cell attachment and spreading of osteoblast-like MG-63 cells seeded on culture plates treated with vitronectin and synthetic peptides. (**A**) Photographs of osteoblast-like MG-63 cells adhering (upper panel) and spreading (lower panel) to culture plates treated with 1% bovine serum albumin (BSA), vitronectin (0.26 μg/cm^2^), scrambled peptide (SP), and VnP-16 peptide (10.5 μg/cm^2^). Bar = 100 μm. (**B**–**C**) Cell attachment (**B**) and spreading (**C**) to immobilized synthetic peptides. MG-63 cells were allowed to adhere to peptide-treated plates for 1 h (**B**) or 3 h (**C**) in serum-free medium. (**D**) The viabilities of osteoblast-like MG-63 cells treated with VnP-16 for 24 or 48 h. ^**^ *p* < 0.01 vs. the SP-treated control group. Data in (**B**–**D**) (*n* = 4) represent the mean ± SD.

Next, to determine whether the effects of the VnP-16 peptide on the cell behavior of HOS cells were similar to those of other human osteoblast-like cells, we used human osteoblast-like MG-63 cells. Similar, but not identical, results were obtained for cell behavior in MG-63 cells. Human plasma vitronectin and VnP-16 peptide strongly promoted cell attachment (Figure 3A upper, B) and spreading (Figure 3A lower, C) in MG-63 cells compared to the BSA or SP control. In addition, the cell attachment and spreading activities of VnP-16 peptide were comparable to those of vitronectin (Figure 3A–C). Similarly, VnP-16 peptide did not affect the proliferation or viability of MG-63 cells (Figure 3D). However, the cell migration activities of vitronectin and the VnP-16 peptide were different between HOS and MG-63 cells. In other words, vitronectin and the VnP-16 peptide had no effect on cell migration in MG-63 cells (data not shown). Therefore, the cellular responses of the VnP-16 peptide to the human osteoblast-like cells HOS and MG-63 had different effects on cell migration but had similar effects on cell attachment, spreading, and viability.

### 3.3. Histomorphometry

**Figure 4:**
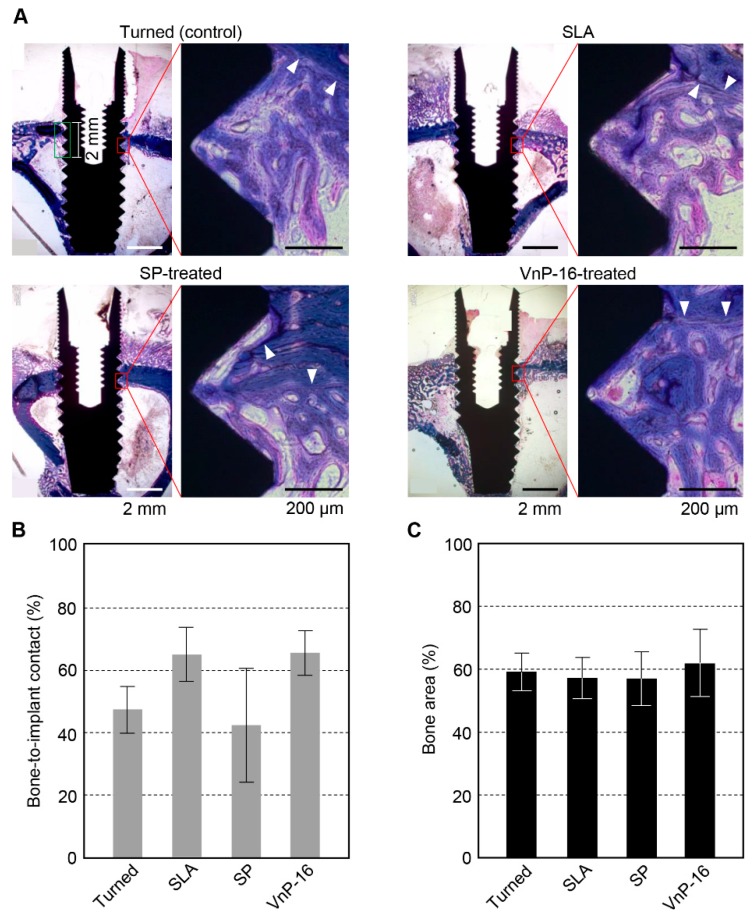
The histologic views and histomorphometric data for bone responses to the turned, SLA, SP-treated SLA, and VnP-16-treated SLA titanium implant surfaces. (**A**) The demarcation lines (white arrowheads), difference in stained colors and maturity of the bone (cancellous or cortical) differentiate the new bone from the existing old bone. Here, the new bone is stained more reddish while the old bone is stained more blueish. (**B**) Bone-to-implant contact ratios were measured, which are defined as the percentage of the implant surface in contact with bone to the total implant surface at the region of interest, which was the area ranging from the bone crest to 2 mm in depth in this study (green edged rectangle in (**A**)). (**C**) The ratio of the area filled with bone to the total area of the region of interest (bone area, or BA ratio) was also measured for each implant.

Every experimental animal was healthy, and no signs of diseases or pathologic states were found until the sacrifice. There were no special inflammatory or immune cells found in the light microscopic view of the specimens. After 14 days of implant insertion, sufficient mineralization was observed in each section (Figure 4A). The mean value and standard deviation of each group were 47.0% ± 7.5% for the turned surface, 64.4% ± 8.6% for SLA, 42.1% ± 18.1% for SP-treated and 65.0% ± 7.2% for the VnP-16-treated surface. The histomorphometric data showed significant differences in BIC (*p* = 0.027). However, the pairwise comparisons between the groups found no significant differences in BIC (Figure 4B). The mean values and standard deviation in the BA were 58.8% ± 6.0% for the turned group, 56.8% ± 6.4% for SLA, 56.6% ± 8.4% for the SP-treated group, and 61.5% ± 10.6% for the VnP-16-treated group (Figure 4C). There were no significant differences in BA among the groups.

## 4. Discussion

The results of these in vitro and in vivo studies indicated that the VnP-16-treated SLA titanium surface augments the initial bone response to a dental implant. The VnP-16 bioactive peptide is expected to accelerate early bone healing further when this peptide is applied to the SLA titanium dental implant. VnP-16 is another candidate biomolecule for stronger osseointegration into a dental implant that is clinically applicable, together with some laminin-derived peptides [3,5,23]. Because VnP-16 has both the upregulation of osteoblast activity and the downregulation of osteoclast activity, different from other peptides, the clinical applicability of this material is considered to be higher [9].

Since VnP-16 downregulates osteoclast activity, this vitronectin-derived peptide is applicable to osteoporotic patients as well as to normal patients. A previous study has already shown the improved bone healing capacity of VnP-16 in an in vivo experiment using ovariectomized rats [9]. The SLA titanium implant is well-known to have long-term clinical performance with high survival rates (higher than 95%) [28,29]. However, this micro-roughened titanium surface has some limitations in the use for patients with a problem in bone metabolism, including osteoporosis [30]. The cell adhesion molecules can potentiate the bone healing capacities of the modified titanium surfaces used in dental clinics without immune responses, which is shown in this study. The effect of VnP-16 on osseointegration of the SLA dental implant needs to be evaluated for normal and osteoporotic patients.

This study used the physical adsorption method for the VnP-16 application to titanium surface. One of the main advantages of this method is the simplicity, that is, an easy process to functionalize the titanium implant surface while this method has several important disadvantages like low effective peptide concentrations, and denaturation of the three dimensional structures of proteins or peptides [19]. The concentration of VnP-16 was determined from a dose-response curve through the cell attachment assay, as in a previous study (data not shown) [9]. The lowest concentration, showing the maximal effect in cell attachment, was used in this study, which solved the problem of low effective peptide concentration. VnP-16 is a peptide composed of 12 amino acids, which are considered to have no specific three dimensional structure [5]. Furthermore, the other approaches to peptide immobilization require additional reactions and costs [19]. Therefore, physical adsorption was the method of choice in this study although more effective approach needs to be investigated continuously.

In the analysis of surface chemistry of this study, higher nitrogen contents on the peptide-treated surfaces imply the applications of the peptides to the titanium implant surfaces. However, the results of the amounts of carbon detected on the surfaces were hard to interpret, despite that carbon was the most abundant on all the surfaces investigated in this study. These unclear results are considered to be from the phenomenon of hydrocarbon contamination on titanium surface, which is usual when titanium is exposed to air [31,32].

Although the analysis of variance test for BIC in this study showed a *p*-value less than 0.05, the pairwise comparisons found no significant differences between the groups. Perhaps, large standard deviation, especially obtained from the measurements of the turned and SP-treated implants, caused no significant differences in the pairwise comparisons. The high mean BIC ratios in the SLA and VnP-16-treated groups and the low mean BIC in the turned and SP-treated groups were considered to contribute to the significant difference in the analysis of variance test for all the groups. The large standard deviation of the data occurred because of the small sample size in this study. Another reason for the large deviation might be the difficulty in displaying the entire three-dimensional bone-implant interface in light microscopic histology. The selection of one cross-sectional plane for light microscopy is arbitrary, and the data from the cross-section are poorly correlated with the data measured on the whole three dimensional image [33]. In order to obtain the similar data between two- and three-dimensional images, three to four histologic sections for each specimen are needed, which are extremely difficult to prepare from a undecalcified specimen, including a titanium implant [34]. Methodological advancements, like three dimensional imaging analysis by micro-computed tomography, and a technique to make more histologic sections from a hard specimen are needed for more obvious in vivo results.

## 5. Conclusions

A human vitronectin-derived peptide, VnP-16 (RVYFFKGKQYWE motif), showed excellent histomorphometric osseointegration data without any special antigen–antibody reaction when this peptide was treated on an SLA titanium dental implant, which has been successfully used in clinics. From the in vitro results of this study, VnP-16 promotes the attachment of osteogenic cells and differentiation into osteoblasts, which may increase the bone healing capacity of the SLA’s titanium surface. Considering both the in vitro and in vivo results of this study, VnP-16 reinforces the osteogenic potential of the SLA titanium dental implant when this peptide is applied to the SLA surface. In the future, VnP-16 may expand the clinical indications of SLA titanium dental implants.
